# The Impact of Aging on Brain Pituitary Adenylate Cyclase Activating Polypeptide, Pathology and Cognition in Mice and Rhesus Macaques

**DOI:** 10.3389/fnagi.2017.00180

**Published:** 2017-06-12

**Authors:** Pengcheng Han, Megan Nielsen, Melissa Song, Junxiang Yin, Michele R. Permenter, Julie A. Vogt, James R. Engle, Brittany N. Dugger, Thomas G. Beach, Carol A. Barnes, Jiong Shi

**Affiliations:** ^1^Department of Neurology, Barrow Neurological Institute, St. Joseph’s Hospital and Medical CenterPhoenix, AZ, United States; ^2^Department of Pathology and Laboratory Medicine Resident Program, Medical University of South CarolinaCharleston, SC, United States; ^3^California National Primate Research Center, University of California, DavisDavis, CA, United States; ^4^Evely F. McKnight Brain Institute, University of ArizonaTucson, AZ, United States; ^5^Institute for Neurodegenerative Diseases, University of California, San FranciscoSan Francisco, CA, United States; ^6^Civin Laboratory for Neuropathology, Banner Sun Health Research InstituteSun City, AZ, United States; ^7^Division of Neural Systems, Memory and Aging, University of ArizonaTucson, AZ, United States; ^8^Departments of Psychology, Neurology, and Neuroscience, University of ArizonaTucson, AZ, United States; ^9^Department of Neurology, Tianjin Neurological Institute, Tianjin Medical University General HospitalTianjin, China

**Keywords:** Alzheimer’s disease, PACAP, aging, nonhuman primates

## Abstract

Pituitary adenylate cyclase activating polypeptide (PACAP) is associated with Alzheimer’s disease (AD), but its age-related effects are unknown. We chose the rhesus macaque due to its closeness to human anatomy and physiology. We examined four variables: aging, cognitive performance, amyloid plaques and PACAP. Delayed nonmatching-to-sample recognition memory scores declined with age and correlated with PACAP levels in the striatum, parietal and temporal lobes. Because amyloid plaques were the only AD pathology in the old rhesus macaque, we further studied human amyloid precursor protein (hAPP) transgenic mice. Aging was associated with decreased performance in the Morris Water Maze (MWM). In wild type (WT) C57BL/6 mice, the performance was decreased at age 24–26 month whereas in hAPP transgenic mice, it was decreased as early as 9–12 month. Neuritic plaques in adult hAPP mice clustered in hippocampus and adjacent cortical regions, but did not propagate further into the frontal cortex. Cerebral PACAP protein levels were reduced in hAPP mice compared to age-matched WT mice, but the genetic predisposition dominated cognitive decline. Taken together, these data suggest an association among PACAP levels, aging, cognitive function and amyloid load in nonhuman primates, with both similarities and differences from human AD brains. Our results suggest caution in choosing animal models and in extrapolating data to human AD studies.

## Introduction

Alzheimer’s disease (AD) is definitively diagnosed by the presence of amyloid plaques, neurofibrillary tangles (NFT) and functional impairment in multiple cognitive domains (Vinters, 2015). Our recent studies report a deficit in the levels of pituitary adenylate cyclase activating polypeptide (PACAP) in human AD brains, suggesting that intrinsic protective factors are involved in AD pathogenesis (Han et al., 2014a,b). The role of PACAP in cellular metabolic function is supported by observations that PACAP protects mitochondrial function in the presence of β-amyloid toxicity (Han et al., 2014b). Consistently, the reduction of PACAP in human AD correlates with both cognitive decline and severity of pathologic markers (Han et al., 2015). A key question is whether aging, as an independent factor reduces PACAP and thus at least partially enhances the vulnerability to AD. Furthermore, is it possible that the reduction of PACAP accelerates aging-associated dysfunction and enters a vicious deteriorating cycle? Previous studies appear to support this vicious cycle hypothesis indirectly. For example, PACAP knockout mice show accelerated retinal aging (Kovács-Valasek et al., 2017). PACAP transportation across the blood brain barrier is reduced in aged mice (Nonaka et al., 2002). Vasoactive intestinal peptide and pituitary adenylate cyclase-activating polypeptide receptor type 1 (VPAC1) is downregulated in multiple regions of aged rat brain (Joo et al., 2005; Lee et al., 2010), suggesting that aging brain is less sensitive to PACAP signaling. On the contrary, PACAP immunoreactive cells appear to be paradoxically high in the aged gerbil hippocampus except mossy fiber zone (Du et al., 2011). However, the mutual interaction of aging factor and PACAP needs further clarification.

Nonhuman primates are evolutionarily close to humans, and like other mammals, do not spontaneously show the full spectrum of neuropathological features characteristic of human AD. On the other hand, macaques do show age-related memory deficits (Rapp et al., 1997) that are reminiscent of those observed in humans (Moffat and Resnick, 2002) and rodents (Barnes, 1979). Additionally, it has been reported that older monkeys can show diffuse amyloid plaques, but rarely show mature NFT. Little is known about what impact these neuropathological markers may have on cognition in these animals.

A majority of transgenic animal models that are used to study AD involve the human amyloid precursor protein (hAPP) gene, which harbors one or more mutations found in familial AD (Cavanaugh et al., 2014). In the J20 mouse model, containing hAPP harboring multiple mutations, amyloid plaques typically appear around 8–10 months of age (Mucke et al., 2000). Preclinical tests of potential drug candidates in this model are usually conducted before 6 months of age when amyloid levels begin to increase, often because experiments that maintain animals to older ages are costly to conduct. Pathologic and cognitive features are less explored in these mice during advanced age even though aging is the most paramount risk factor associated with AD. An understanding of what makes an aging brain more susceptible to such pathological conditions is critical for the design of preclinical studies that aim to prevent or delay disease progression (Franco and Cedazo-Minguez, 2014). While an ideal model of AD is not available (Laurijssens et al., 2013; Bilkei-Gorzo, 2014; Franco and Cedazo-Minguez, 2014), further characterization of the brains of normal aging mice and nonhuman primates, along with a mouse model of AD, allows us to define aspects of brain aging that are expected to occur over the lifespan and identify those that do or do not resemble the human disease profile (Cavanaugh et al., 2014). In particular, it will be highly informative to compare the same spectrum of pathologic, biochemical and behavioral parameters among normal and pathologic aging models from different species. This comparison will point out key factors underlying common aging physiology and pathology. Here we characterize cognitive performance and PACAP protein in nonhuman primates ranging in age equivalent to human ages from 24 years to 96 years. We further investigated cognitive performance, amyloid plaques, and PACAP protein in hAPP transgenic mice and compared to their wild type (WT) littermates that were in an age range equivalent to 40–80 human years, within the age range of onset of both familial and sporadic AD (Friedland et al., 1988). We aim to compare these key AD features in the transgenic mice during aging, and to compare them with that in human AD.

## Materials and Methods

### Animals

Twenty-four rhesus macaques (*Macaca mulatta*) were included in this study, 15 males and 9 females. These animals ranged in age from 8 years to 32 years (Supplementary Table S1). These ages can be multiplied by a factor of 3 to provide an approximate comparison to human aging (Tigges et al., 1988) of approximately 24–96 years. The animals were housed in pairs and were provided with environmental enrichment, including socialization in a large pen, behavioral testing and regular fruits and vegetables. These monkeys were all born and housed continuously at the California National Primate Research Center in Davis, CA, USA and were part of a long term study of the effects of aging on the primate temporal lobe. At the end of the data collection for that experiment, animals were euthanized with an overdose of sodium pentobarbital (60 mg/kg, intravenously), and transcardially perfused with a solution of 4% paraformaldehyde in 0.01% phosphate-buffered saline (pH 7.4), followed by a mixture of 4% paraformaldehyde and 10% sucrose. After perfusion, the brains were extracted and placed into a solution of 4% paraformaldehyde and 30% sucrose for further cryoprotection. The entire brain of each monkey was serial sectioned coronally at 30 μm while frozen on a sliding microtome. A one in four section series was Nissl stained so that sections of interest in the frozen material could be more easily identified from the archived brains for future studies, such as the present one (Thome et al., 2016). All procedures on rhesus macaques followed an Animal Care and Use protocol approved by the IACUC at the University of California, Davis, CA, USA.

The hAPP transgenic mice (B6.Cg-Tg (PDGFB-APPSwInd) 20 Lms/2Mmjax, stock number 006293/J20) were generated as described previously (Mucke et al., 2000). The breeding pair was purchased from The Jackson Laboratory (Bar Harbor, ME, USA). This strain harbors hAPP with Swedish (K670N/M671L) and the Indiana (V717F) mutations. The C57BL/6 WT mice were used as littermate controls. The breeding pair was housed in the Barrow Neurological Institute (BNI) vivarium and the offspring were genotyped using the protocol supplied by the vendor. A total of 87 mice (51 WT, 36 hAPP) were included in this study with a total mortality of 12% during the experiments (WT mortality 8%, hAPP mortality 17%). Individual animals may be used for measuring multiple parameters. The procedures in all hAPP transgenic mice were consistent with NIH Animal Care and Use guidelines and were approved by the Institute Animal Care and Use Committee (IACUC) at University of California, Davis, CA, USA and BNI.

### Cognitive Test for Rhesus Macaques

The Delayed Nonmatching-to-Sample Task (DNMS) was conducted in the Wisconsin General Testing Apparatus as described previously (Shamy et al., 2006). The first component of the task was to present the “sample object” in the middle of three wells in front of the monkey. The monkey could displace this object during this phase of the task to receive food reward. After varying delays (increasing from 10 s to 15 s, 30 s, 60 s and 10 min), the monkey was given a choice of two objects that cover the outer wells. One object was the sample object and the other was novel. A correct response was to displace the novel object to receive food reward (this is the nonmatching rule). The delay interval utilized for the present analysis was 10 min because it was the longest delay tested, and thus the most challenging for the older animals. The behavior scores were calculated to represent the percentage of correct responses at that delay interval. The interval between cognitive testing and euthanasia typically ranged from 6 months to 3 years.

### Histopathological Assessment of Plaque Density and Neuronal Expression of PACAP in Rhesus Macaque Brains

Thirty micron free floating coronal sections were stained with thioflavin S and enhanced silver methods for amyloid plaques (Braak and Braak, 1991). In addition, Immunohistochemistry (IHC) was done with an antibody directed against amino acid residue 1–16 of beta amyloid (6E10, BioLegend, San Diego, CA, USA). Amyloid plaque density was graded and staged at standard sites in frontal, temporal, parietal and occipital cortices as well as hippocampus and entorhinal cortex, based on the aggregate impression from the 30 μm stained sections. Neuritic plaque density scores were obtained by assigning values of none, sparse, moderate and frequent, according to the published CERAD templates (Mirra et al., 1991). Based on studies of human brain (Han et al., 2014a, 2015), PACAP is notably expressed in the hippocampus, temporal lobe, striatum, and parietal lobe. We selected samples of these four areas for analysis using the Paxinos Atlas, Plate 70. The slide was stained with primary antibodies (rabbit-anti-PACAP, Santa Cruz Biotechnology, Dallas, TX, USA; mouse-anti-NeuN, Millipore, Billerica, MA, USA), and then washed and stained with secondary fluorescent antibodies. Omission of primary antibody was performed as a negative control and human brain tissue was used as a positive control. Images were taken using a Zeiss LSM 510 confocal microscope. The PACAP (green) channel gain was uniformly set at 450 and the NeuN nuclear (red) channel gain was set at 500. In each anatomic site, three representative images were taken at equidistance in a linear dimension: one image represents central area and two images represent paracentral areas. On each image, 10 or more NeuN positive neurons were randomly selected and outlined as Region of Interest (ROI) for analysis. The PACAP fluorescence intensity in the defined ROI was recorded (>30 neuronal ROIs for each image) and averaged. All analyses were conducted blinded to the animal’s identity, age, or other relevant information.

### Morris Water Maze (MWM) for Mice

MWM testing was performed according to the protocol by Morris (Morris, 1984) and as previously carried out in our lab (Yin et al., 2011). Briefly, MWM tank (diameter = 1.5 m) was divided into four quadrants at equal distance to the outer perimeter. The water temperature was maintained at 24°C. The water was made opaque by adding 300 g non-toxic white tempera powder (Jack Richeson and Co. Inc., Kimberly, WI, USA). Each mouse was allowed to swim for 120 s and the time spent to reach the platform (escape latency) was recorded in each trial during the training period (target-on training). Each mouse had 16 training trials divided equally during a 4-days period. In each trial, mice that successfully located the platform were allowed to stay on the platform for 10 s, whereas those that failed to locate the platform within the defined time frame were placed on the platform for 10 s by the experimenter. A single probe trial was conducted by removing the platform (target-off test) on the 5th day, in which all mice started from the farthest point opposite to the original target platform location and were allowed to swim for 120 s. We used the EthoVision 3.1 tracking system (Noldus Information Technology Inc., Leesburg, VA, USA) to record and analyze the data.

### Two-Object Novel Object Recognition (NOR) for Mice

NOR task was conducted in an open field arena with two different types of objects (Hammond et al., 2004; Ennaceur, 2010). Both objects were generally consistent in height and volume, but different in shape and appearance. During habituation, the animals were allowed to explore an empty arena. Twenty-four hours after habituation, the animals were exposed to the familiar arena with two identical objects placed at an equal distance from the arena walls. The next day, mice were allowed to explore the open field in the presence of the familiar object and a novel object to test their long-term memory. The time spent in exploring each object was recorded. The discrimination index (DI) was calculated as:

$$DI=\frac{\text{Time exploring new object }-\text{ Time exploring old object}}{\text{Total object exploring time}}$$

### Amyloid Plaque Stain for Mice

Mice were deeply anesthetized with isoflurane and transcardiacally perfused with 0.1 M phosphate-buffer saline (PBS). Brains were harvested and dissected on an ice plate. Left hemispheres were fixed with 10% formalin (Thermo Scientific Inc. Waltham, MA, USA) at 4°C for at least 72 h. Right hemispheres were immediately frozen in liquid nitrogen and stored in −80°C for biochemical assays (see “PACAP Elisa in Mice” Section). The formalin fixed left hemispheric brain tissues were embedded in paraffin blocks, coronally sectioned at 6 μm thickness (between Bregma ±2.5 mm, [Fig F1]Figure 2A), and mounted on Silane-coated glass microscope slides (Sigma, St. Louis, MO, USA). For staining amyloid plaques, slides were deparafinized in xylene and ethanol in the following series of procedures: 100% xylene for 5 min twice, xylene/ethanol mix (1:1) for 5 min, 100% ethanol 5 min, 95% ethanol 5 min, 70% ethanol 5 min, 50% ethanol 5 min, and water for 5 min twice. Then the slide was incubated in filtered 1% aqueous thioflavin-S (Sigma-Aldrich, St Louis, MO, USA) for 10 min at room temperature in a light-proof box. After two washes in 80% ethanol for 5 min followed by a wash in 95% ethanol for 5 min, the slides were rinsed thoroughly in distilled water. The slides were then sealed with coverslips and stored at 4°C.

**Figure 1 F1:**
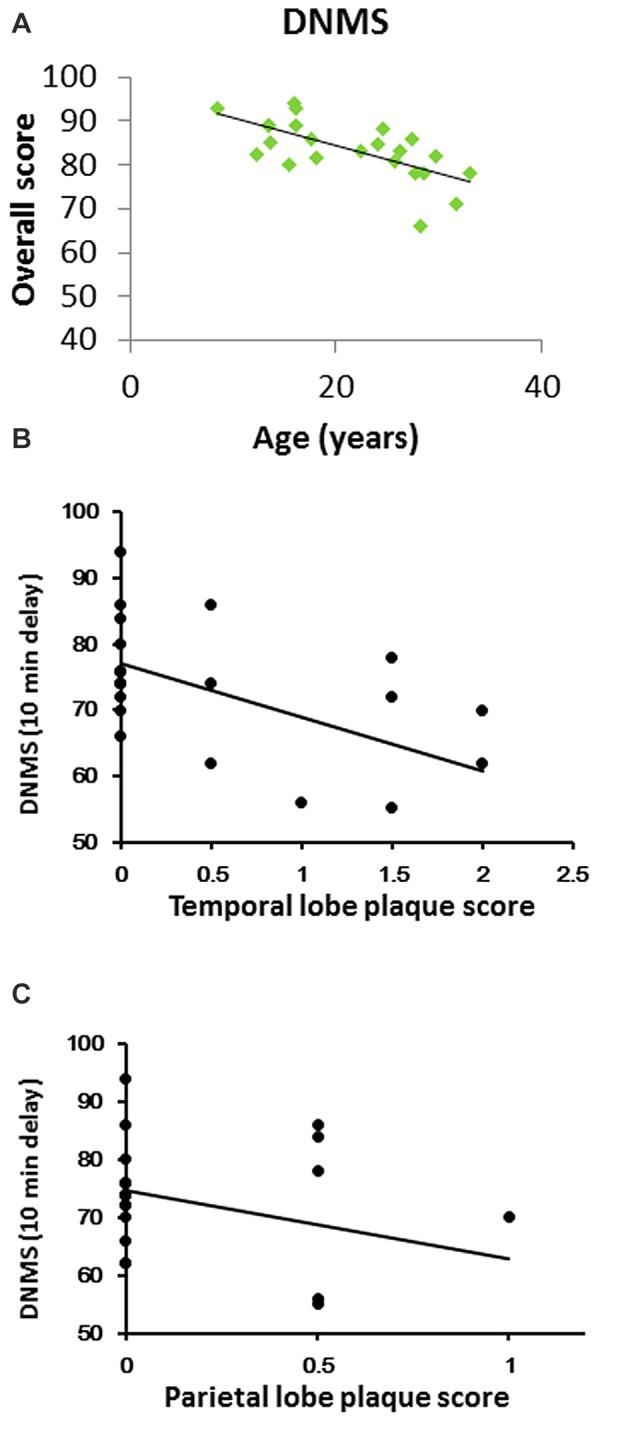
Cognitive performance in a delayed nonmatching-to-sample (DNMS) task across age in rhesus macaques. **(A)** DNMS performance (percent correct at a 10 min delay) declined linearly throughout the lifespan (Pearson *R* = −0.67, *P* < 0.001). **(B)** DNMS performance inversely correlated with temporal lobe plaque density (CERAD plaque score). **(C)** DNMS inversely correlated with parietal lobe plaque density.

**Figure 2:**
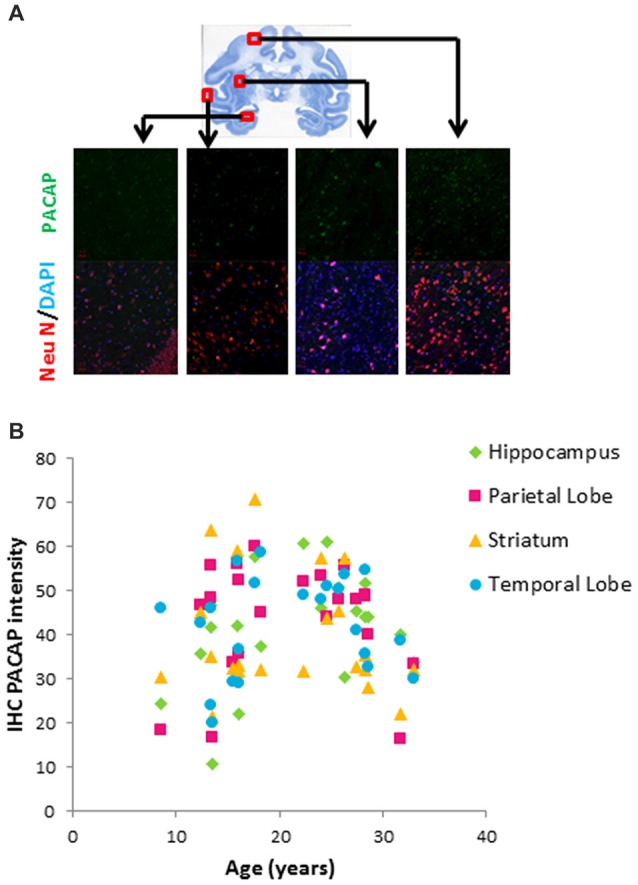
Age-related changes in pituitary adenylate cyclase activating polypeptide (PACAP) in different brain areas of rhesus macaques. **(A)** Immunohistochemistry (IHC) of PACAP. Double staining of NeuN (red) and DAPI (blue) depicted neurons. Immunostaining intensity of PACAP (green) was assessed within the randomly selected 10 neurons in each visual field. At least three visual fields were assessed for each slide and averaged to calculate IHC intensity of PACAP for each individual animal. PACAP intensity from each region (hippocampus, parietal lobe, striatum and temporal lobe) was plotted against age. **(B)** Biphasic pattern of PACAP across the life span.

### PACAP Elisa in Mice

Frozen mouse brain tissues obtained from the hippocampal formation and adjacent cortices (area indicated in Figure 2A inset) were homogenized in RIPA buffer supplemented with a proteinase inhibitor cocktail (Sigma-Aldrich, St Louis, MO, USA). Protein samples were quantified for PACAP using a standard enzyme-linked immunoassay kit (MBS720617, MyBiosource Inc., San Diego, CA, USA) according to the manufacturer’s protocol. Briefly, protein samples and the standard PACAP were loaded and incubated with a biotin-labeled PACAP antibody at 37°C for 60 min. The plate was washed with the washing buffer 5 times, followed by incubation with chromogen solution at 37°C for 10 min. The reaction was stopped by adding a stop solution. The final colorimetric density was measured at a wavelength of 450 nm using an INFINITE M200Pro plate reader (Tecan systems Inc., San Jose, CA, USA). Meanwhile, the total protein quantity was determined with a Pierce bicinchoninic acid protein assay kit (catalog #23227, Thermo Scientific, Waltham, MA, USA). To eliminate the confounding factor of potential neurodegeneration, we normalized the PACAP level to the measured protein level in brain tissues. Therefore, PACAP in cortical tissues was expressed as nanograms per milligram of total protein.

### Data Analysis and Statistics

Correlation coefficients were examined using a Spearman linear model unless indicated otherwise. If the linear model did not fit well, we further tested nonlinear models (exponential, sigmoid and polynomial) and chose the model that best fit the data points, as judged by the largest *r*^2^ (goodness of fit). We use one way ANOVA with *post hoc* Tukey tests to compare mean values of multiple groups, or two-way ANOVA with *post hoc* Bonferroni tests if two intrinsic factors (age and genetic background) are involved. A *p* value less than 0.05 was considered to be statistically significant.

## Results

We examined cognition, plaques and PACAP expression in rhesus monkeys in the age range from 8 years to 32 years old. This age range is equivalent to 24–96 years old in a human life span. A rhesus monkey that is 20 years old is equivalent to a 60 year old human. Therefore we define those 20 years old or less as the young group and those above 20 years as the old group.

We tested DNMS performance at a long delay (10 min), and it progressively decreased with increasing age (*r* = −0.670, *P* = 0.0003, Figure 1A).

Plaques were evident in the temporal and inferior parietal lobes in most of the old rhesus monkeys. Of the areas analyzed, the temporal lobe had the highest plaque density. Furthermore, DNMS performance inversely correlated with both temporal and parietal lobe plaque density (Figure 1B: *r* = −0.609, *p* = 0.002; Figure 1C: *r* = −0.547, *p* = 0.007). Of note, only two (both aged 28 years old) showed amyloid plaques in the hippocampus. There was no evidence of NFT in the brain.

We further characterized neuronal expression of PACAP using a double staining immunohistochemical approach (Figure 2A). While this approach is not as quantitative as ELISA, it provides a semi-quantitative assessment specific to the neuronal population. Within the four regions we examined (hippocampus, parietal lobe, striatum and temporal lobe), neuronal expression of PACAP showed a biphasic pattern; it increased with age in the young group and then declined with age in the old group (Figure 2B).

**Figure 3:**
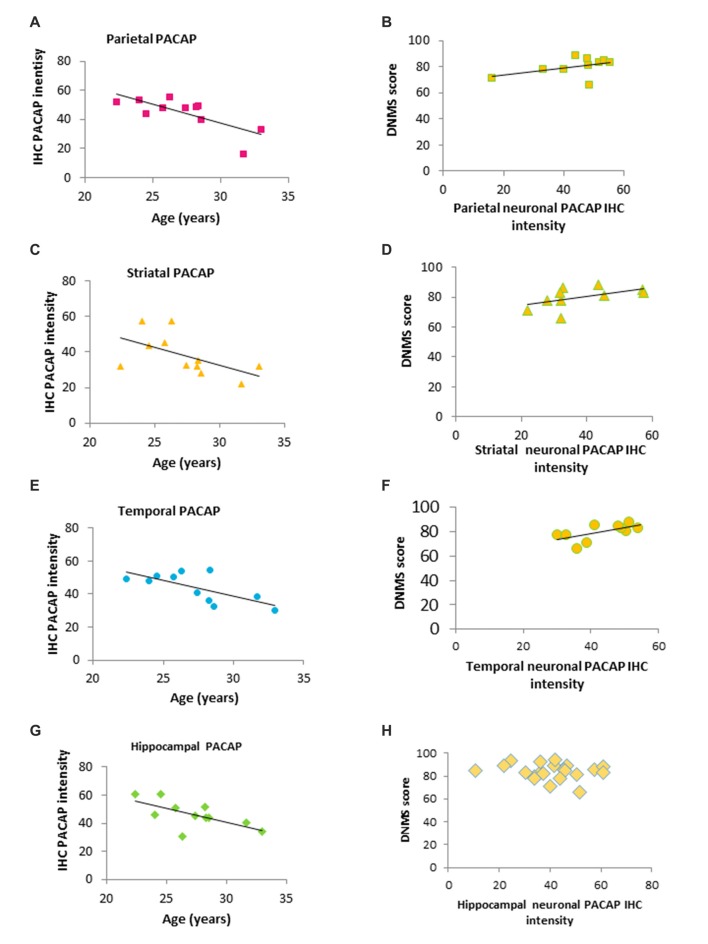
Correlation of age, PACAP and DNMS performance in old rhesus macaques. **(A,C,E,G)** Regional IHC intensity of neuronal PACAP in the old group of rhesus macaques (20–32 years). PACAP levels in all four regions (the hippocampus, parietal lobe, striatum and temporal lobe) declined with age progression. **(B,D,F,H)** Higher levels of regional PACAP correlated with better performance on the DNMS task in the old group of monkeys in Parietal **(B)** Striatal **(D)** and Temporal lobe **(F)** while no correlation was found in the hippocampus **(H)**.

In the old group (≥20 year-old), neuronal expression of PACAP in all four regions were inversely correlated with advanced age (20–32 years; Parietal: Figure 3A, *r* = −0.743, *p* = 0.000028; Striatal: Figure 3C, *r* = −0.556, *p* = 0.0046; Temporal: Figure 3E, *r* = −0.686, *p* = 0.00019; Hippocampal: Figure 3G, *r* = −0.651, *p* = 0.00054). Furthermore, DNMS performance in the older animals showed a positive correlation with PACAP intensity. In parietal, striatal, and temporal lobes, the higher the neuronal expression of PACAP was, the better the performance on the DNMS test. This did not hold true for hippocampal PACAP (Parietal: Figure 3B, *r* = 0.469, *p* = 0.020; Striatal: Figure 3D, *r* = 0.545, *p* = 0.005; Temporal: Figure 3F, *r* = 0.136, *p* = 0.001; Hippocampal: Figure 3H, *r* = −0.023, *p* = 0.105).

Since amyloid plaques were the only AD like pathology present in rhesus monkeys, we used hAPP mice for further studies. Human APP mice are reported to show memory deficits as early as 4–5 months (Wright et al., 2013) or 6–7 months (Palop et al., 2003), as measured by different maze paradigms. We tested hAPP mice at age groups of 9–12 months, 13–16 months and 24–26 months. Given that the mean lifespan of a B6 mouse is approximately 800 days (Goodrick, 1975; equivalent to 26.7 months), the group of 24–26 months old mice is approximately equivalent to 80 human years. The age group of 9–12 months is equivalent to 40 human years; this is when clinical symptoms of familial AD appear. The age group 13–16 months is equivalent to 60 human years, when sporadic AD onset begins to increase, and memory may decline in the normal aging human population.

**Figure 4:**
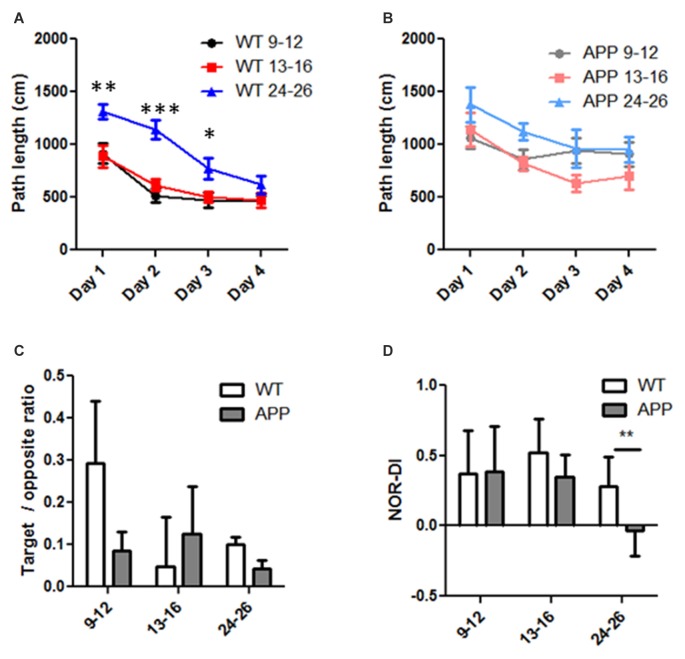
Age and genetic effects on Morris Water Maze (MWM) and Novel Object Recognition (NOR) performance in human amyloid precursor protein (hAPP) and wild type (WT) mice. **(A,B)** Path length to reach the platform was progressively reduced during the 4-days training course in WT **(A)** and hAPP **(B)** mice. Note the reduction was less in hAPP **(B)** than that of WT **(A)** mice. **(C)** In the probe test, the ratio of path length in the target quadrant and the opposite quadrant were compared in the three age groups in WT and hAPP mice. **(D)** The discrimination index (DI) in the NOR test was compared in the three age groups and in hAPP and WT mice. For all experiments in **(A–C)**, two-way ANOVA and *post hoc* Bonferroni tests were applied. *Indicates *p* < 0.05, **indicates *p* < 0.01, ***indicates *p* < 0.001.

We performed the MWM tests on each group. Learning is indicated by reduced path lengths as training progressed. All age groups in WT mice showed successful learning (Figure 4A). Based on two-way repeated ANOVA, the age factor contributed to 17% of the total variation (*F* = 27.91, *p* < 0.0001), training days contributed to 24% of the total variation (*F* = 26.48, *p* < 0.0001), and there was no interaction between the training factor and age factors. While learning improvement was evident in all age groups, the oldest age group (24–26 months) performed relatively poorly in the first 3 days compared to the youngest group (9–12 months, *post hoc* Bonferroni test, *p* < 0.01 in day1, *p* < 0.001 in day2 and *p* < 0.05 in day 3). In hAPP mice, the age factor contributed to 5.8% of the total variation (*F* = 3.812, *p* < 0.05), the training factor contributed to 8.9% of variation (*F* = 3.893, *p* < 0.05), and there was no interaction between the two factors (*F* = 0.433, *p* = 0.85, two way repeated ANOVA, Figure 4B). However, *post hoc* analysis did not suggest significant learning improvement over the 4 days training period in any age group (*p* > 0.05, Figure 4B).

In the probe test in which the platform was removed, we calculated the ratio of the swim path length within the target quadrant and that in the opposite quadrant. The hAPP mice have an average ratio of 0.09 ± 0.04 in the 9–12 months group (*n* = 17), 0.13 ± 0.11 in the 13–16 months group (*n* = 8), and 0.04 ± 0.01 in the 24–26 months group (*n* = 5). The mean ratio in WT mice was 0.29 ± 0.14 in the 9–12 months group (*n* = 19), 0.05 ± 0.11 in the 13–16 months group (*n* = 16), and 0.10 ± 0.02 in the 24–26 months group (*n* = 12). Neither the age factor (*F* = 0.502, *p* = 0.608) nor the genetic factor (*F* = 0.359, *p* = 0.551) contributed significantly to the total variation; and there was no significant interaction between these factors (*F* = 0.625, *p* = 0.538, two way ANOVA, Figure 4C). The DI obtained from the NOR tests in hAPP mice did not differ from that of WT except in the 24–26 months group (Figure 4D). The hAPP mice have an average DI of 0.39 ± 0.28 in the 9–12 months group (*n* = 17), 0.35 ± 0.16 in the 13–16 months group (*n* = 8), and −0.03 ± 0.18 in the 24–26 months group (*n* = 5). The mean DI in WT mice was 0.37 ± 0.31 in the 9–12 months group (*n* = 19), 0.52 ± 0.24 in the 13–16 months group (*n* = 16) and 0.28 ± 0.21 in the 24–26 months group (*n* = 1 2). Neither the age (*F* = 0.486, *p* = 0.617) nor genetic (*F* = 0.313, *p* = 0.578) factors made a significant contribution to the total variance, nor was there a significant interaction (two-way ANOVA, interaction = 0.65% of total variation, *P* = 0.925). One exception was that in the 24–26 months age only, NOR-DI of hAPP mice was significantly lower than that of WT (*p* < 0.05, *T*-test, Figure 4D).

**Figure 5:**
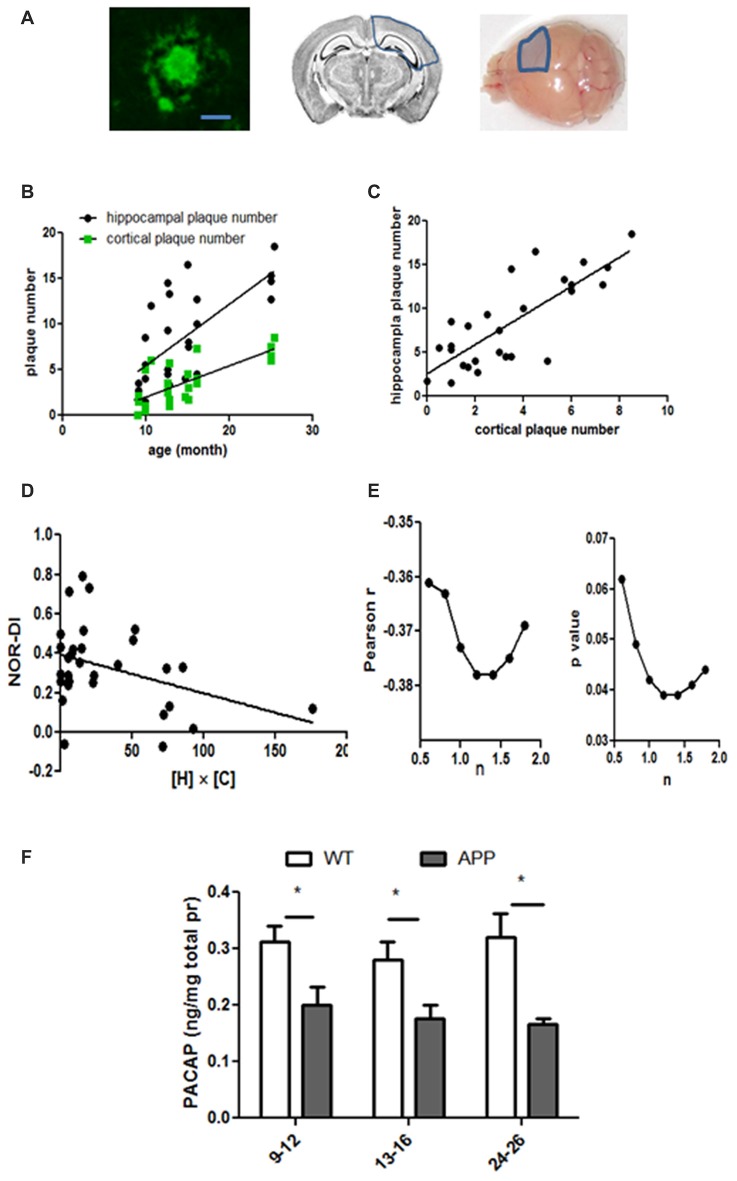
Amyloid plaque numbers in hAPP mice and their relation to age, MWM and NOR performance. **(A)** The left panel shows a typical plaque in hAPP mice (bar length = 20 μm). These plaques were restricted to the hippocampus and adjacent cortices. These areas were outlined by the blue lines in the middle (coronal view) and right (surface view) panels. Only one hemisphere was marked but the plaques were expressed on both sides. **(B)** Plaque numbers in the hippocampus and the cortex increased with age progression. Hippocampus: *r^2^* = 0.48, *p* < 0.0001; cortex: *r*^2^ = 0.55, *p* < 0.0001. **(C)** The hippocampal and cortical plaque numbers correlated with each other (*r*^2^ = 0.63, *p* < 0.001). **(D)** NOR-DI inversely correlated with the product of hippocampal and cortical plaque numbers ([H] × [C]), Pearson *r* = −0.37, *p* = 0.042. **(E)** NOR-DI linearly correlated with *n*. While the linear correlation existed when *n* = 1 **(E)** the correlation remained valid (*p* < 0.05) when *n* was in the range of 0.8–1.8. The correlation was the strongest when *n* was between 1.2 and 1.4. **(F)** Cerebral PACAP levels were measured in three age groups of hAPP and WT mice. The cerebral tissues only included the hippocampus and the cortex around hippocampus (indicated as in the right panel of **A**). Two-way ANOVA and *post hoc* Bonferroni tests were applied. *Indicates *p* < 0.05.

Next, we examined neuritic plaques on the serial coronal sections made from brains of WT and hAPP mice (Figure 5A). At the age of 9-months and older, all hAPP mice showed thioflavin-S positive plaques in the hippocampus. A majority of them also showed thioflavin-S positive plaques in cortices adjacent to the hippocampus (the same coronal section, Figure 5A). However, no thioflavin-S positive plaques were found in frontal cortex or any other locations beyond the coronal sections covering the hippocampal region. Thus, the amyloid plaque distribution in hAPP mice does not resemble the typical pattern seen in human AD brain. Even in the oldest hAPP mice (24–26 months), the plaques did not propagate throughout the brain. In WT, no thioflavin-S positive plaques were seen regardless of age. Plaques within both hippocampus and cortex increased in numbers with age in hAPP mice (hippocampus: *r*^2^ = 0.478, *p* < 0.0001; cortical: *r*^2^ = 0.545, *p* < 0.0001, Figure 5B). The hippocampal and cortical plaque numbers correlated with each other (*r*^2^ = 0.627, *p* < 0.001, Figure 5C), although in each individual animal the cortical plaque numbers were far less than were the hippocampal plaque numbers (Figures 5B,C). NOR-DI did not correlate individually with hippocampal or cortical plaque numbers (data not shown). This may not be surprising because NOR performance is influenced by both hippocampal and cortical lesions (Antunes and Biala, 2012). Because both hippocampal and cortical regions contributed to NOR performance, we multiplied these two independent factors to get the product of hippocampal and cortical plaque numbers ([H] × [C]), and fit NOR-DI with this product. NOR-DI inversely correlated with the product of hippocampal and cortical plaques (*r* = −0.373, *p* = 0.042, Figure 5D). Because we did not know whether there were unequal contributions from these two factors, we systematically varied the relative weight as follows: we modified the product as [H]*^n^*[C]^1^, where [H] denoted the hippocampal plaque number, [C] denoted the cortical plaque number, and n was a factor reflecting the relative contribution of [H] vs. [C]. By varying *n*, we found that NOR-DI correlated best with [H]^*n*^[C] when *n* is in the range of 1.2–1.4 (Figure 5E).

PACAP is reduced in brains of AD patients (Han et al., 2015) and 18-month old 3×Tg AD mice (Han et al., 2014b), so we measured cerebral PACAP levels in different age groups of hAPP mice (Figure 5F). The tissue taken for PACAP assessment was restricted to the hippocampus and adjacent cortices (the right panel, Figure 5A). The average cerebral PACAP was 0.20 ± 0.031 ng per mg total protein in the 9–12 months group (*n* = 9), 0.18 ± 0.023 in the 13–16 months group (*n* = 6), and 0.17 ± 0.009 in the 24–26 months group (*n* = 5). The mean PACAP level in WT mice was 0.31 ± 0.03 in the 9–12 months group (*n* = 18), 0.28 ± 0.031 in the 13–16 months group (*n* = 14), and 0.32 ± 0.042 in the 24–26 months group (*n* = 8). Analyzed by two-way ANOVA, the age factor did not significantly contribute to the variation (0.79% total variation, *F* = 0.283, *p* = 0.755), but the genetic factor did (23% total variation, *F* = 16.56, *p* = 0.0002). This suggests hAPP genetic mutation overshadows the age factor and dictates the expression levels of PACAP. Thus, the age factor does not account for varying levels of PACAP in WT mice. However, the genetic factor (hAPP) significantly decreased PACAP in each age group compared with the same age WT mice (*p* < 0.05 in each age group, two-way ANOVA with *post hoc* Bonferroni tests, Figure 5F).

## Discussion

Recent longitudinal studies reconfirm that cognitive performance declines as age increases in AD but also, to a lesser degree, in individuals without AD pathological features (Monsell et al., 2014; Jack et al., 2015). However, in a subpopulation of normal cognitive individuals, amyloid plaques can be evident, albeit less severe than that of Alzheimer’s patients. Patients without amyloid plaques tend to have better cognitive performance scores, although patients with amyloid plaques do not necessarily have worse cognition (Kawas et al., 2015), suggesting additional protective factors may sustain normal cognition. Although the amyloid plaque number inversely correlates with premortem cognition, NFT correlate better (Nelson et al., 2012). Among the various candidate protective factors, PACAP levels significantly correlate with cognitive performance and inversely correlate with amyloid plaques (Han et al., 2015).

The older rhesus macaques examined here did exhibit amyloid plaques in the brain. Most areas, however, contained very low plaque densities, whereas the temporal lobe had the highest density. Advanced age is a significant predictor of these deposits: the older monkeys in this cohort were more likely to contain plaques (*r* = 0.577, *p* < 0.01). Therefore, the aging primate model and the non-AD aging human show some similarities, where sparse to moderate amyloid plaques are often detected at autopsy and increased with age. In the cognitive domain, DNMS performance progressively declines with the aging process (20–32 years, equivalent to 60–96 human years). Importantly, amyloid plaque density, although not as heavy as observed in human AD, correlates with a decline in cognition, as assessed by the DNMS task, although this effect is not separable from the effect of aging alone. Similar to humans, neuronal PACAP levels in the macaque also correlate with cognitive function in the older monkeys. Because PACAP levels were previously measured only in older human brains (>65 years), the levels in young human brain is unknown, but may show a rise during aging as is observed here in the monkey. It would be interesting to determine whether a similar age-dependent PACAP decline exists in healthy human populations as is observed here in older rhesus macaques.

Rodent AD models are commonly used to authentically reflect the primary features of the disease. We characterized these features in hAPP mice that were in an age range equivalent to the human age of 40–80 years. We show here that MWM performance decreases with age in WT mice, while in hAPP mice performance was equally poor in all age groups older than 9 months, suggesting that by middle age this genotype already has cognitive impairments. While these relationships are similar to that in the familial AD population, unlike human AD patients, amyloid plaques in hAPP mice are restricted to the hippocampus and adjacent cortices. It is unclear why amyloid plaques in mice do not form or propagate to multiple regions, as the APP-driving PDGFB transcription factor is widely expressed in neurons throughout the rodent brain. Because of the temporal lobe distribution of plaques, the plaque numbers were correlated with hippocampus-based behavioral tasks such as the MWM. Although mice with extensive plaque deposition showed impaired performance on the MWM, MWM performance was not linearly correlated with amyloid plaque deposition. Non-linear relationships have been observed in early clinicopathologic study in human AD (Blessed et al., 1968), but a more recent meta-analysis predicts a linear relationship between the amyloid plaque number and cognitive performance in humans (Nelson et al., 2012). Thus, the relationship between plaque burden and cognition in the hAPP mice in the present study differs from human AD observations. This implies that potential protective factors may not be sufficient to retain cognition in the face of the imposed genetic predisposition for plaque formation in this model. The high dosage of amyloid clusters that are localized in mouse hippocampus may be so strong that it overpowers such protective factors. While PACAP reduction in human AD inversely correlates with amyloid plaques and cognitive decline, brain PACAP levels in mice did not correlate with plaque numbers or water maze performance (data not shown). The hAPP mouse model was designed to model familial AD, but not sporadic AD. The reduced cerebral PACAP in hAPP mice is driven by hAPP gene rather than the age factor, thus it does not resemble human studies where cerebral PACAP is closely correlated with both cognitive function and pathologic markers (Han et al., 2015).

In conclusion, nonhuman primates show an age-related cognitive decline that is closely associated with both plaque accumulation and cerebral PACAP reduction. For hAPP mice, however, genetic factors appear to dominate PACAP reduction and cognitive decline. Our results highlight the importance of choosing animal models that are optimized for obtaining answers to a given experiment’s specific question, when searching for a deeper understanding of the disease.

## Author Contributions

PH: design of the study, acquisition and analysis of data and drafting the manuscript and figures. MN, MS, JY, MRP, JAV, JRE and BND: acquisition of data and revising the manuscript and figures. TGB: analysis of data and revising the manuscript. CAB: conception of the study, analysis of data and revising the manuscript and figures. JS: conception and design of the study, analysis of data and revising the manuscript and figures.

## Conflict of Interest Statement

The authors declare that the research was conducted in the absence of any commercial or financial relationships that could be construed as a potential conflict of interest.
