# Supplementary material for: The Impact of Aging on Brain Pituitary Adenylate Cyclase Activating Polypeptide, Pathology and Cognition in Mice and Rhesus Macaques
Source: Front Aging Neurosci. 2017 Jun 12;9:180. doi: 10.3389/fnagi.2017.00180 (PMC5467357; doi:10.3389/fnagi.2017.00180)
Supplement: Supplementary file 1 [file Table_1.docx]

Supplementary table: Gender and age of rhesus macaque in the young and old groups.

| **gender** | **group** | **age (years)** |
| --- | --- | --- |
| male | young | 8.46 |
| male | young | 12.36 |
| male | young | 13.42 |
| male | young | 13.42 |
| male | young | 13.54 |
| male | young | 15.97 |
| male | young | 16.09 |
| male | young | 17.58 |
| female | young | 15.42 |
| female | young | 16.03 |
| female | young | 18.14 |
| female | old | 24.55 |
| female | old | 25.75 |
| female | old | 27.42 |
| female | old | 28.33 |
| female | old | 31.67 |
| female | old | 32.97 |
| male | old | 22.36 |
| male | old | 24.00 |
| male | old | 26.29 |
| male | old | 27.67 |
| male | old | 28.23 |
| male | old | 28.58 |
| male | old | 29.67 |
